# Supplementary figures and images for: Targeted RNAseq Improves Clinical Diagnosis of Very Early-Onset Pediatric Immune Dysregulation
Source: J Pers Med. 2022 Jun 1;12(6):919. doi: 10.3390/jpm12060919 (PMC9224647; doi:10.3390/jpm12060919)

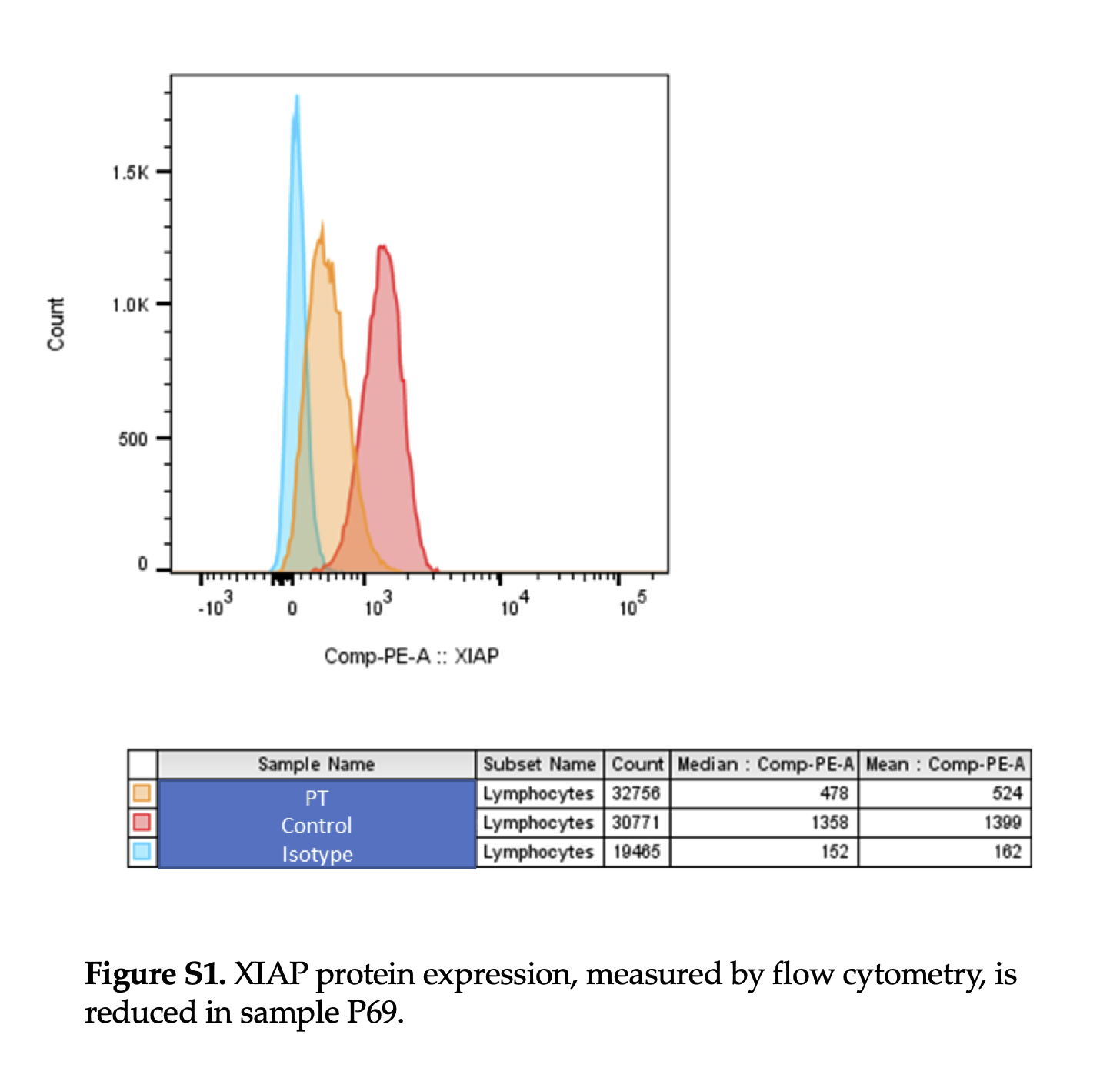

Supplement: Supplementary file 1 [file jpm-12-00919-s001.zip › Figure S1.tiff]
